# Investigating the clinical implication of corneometer and mexameter readings towards objective, efficient evaluation of psoriasis vulgaris severity

**DOI:** 10.1038/s41598-022-11573-2

**Published:** 2022-05-06

**Authors:** Chao-Kai Hsu, Nan-Yu Cheng, Chao-Chun Yang, Yun-Yo Yen, Sheng-Hao Tseng

**Affiliations:** 1 Department of Dermatology, College of Medicine, National Cheng Kung University Hospital, National Cheng Kung University, Tainan, 701 Taiwan, ROC; 2 International Center for Wound Repair and Regeneration, National Cheng Kung University, Tainan, 701 Taiwan, ROC; 3 Department of Optometry, Shu-Zen College of Medicine and Management, Kaohsiung, 821 Taiwan, ROC; 4 Department of Photonics, National Cheng Kung University, Tainan, 701 Taiwan, ROC; 5 School of Dentistry, College of Dental Medicine, Kaohsiung Medical University, Kaohsiung, 807 Taiwan, ROC

**Keywords:** Near-infrared spectroscopy, Skin diseases

## Abstract

In clinical settings, although Psoriasis Area and Severity Index (PASI) scoring system can provide a quick visual assessment of the severity of psoriasis vulgaris, there is still a strong demand for higher efficiency and accuracy in quantifying the inflammation status of psoriatic lesions. Currently, there are already commercial systems, such as the Courage + Khazaka Corneometer and Mexameter that measure skin capacitance and optical reflectance, for conveniently quantifying the status of skin barrier function and erythema of skin. Despite numerous comparisons of the Courage + Khazaka system with the PASI scoring system, they are rarely compared on parity with diffuse reflectance spectroscopy (DRS) based systems. In this study, we employed a custom-built DRS system shown to be able to determine the skin water-protein binding status and the hemoglobin concentration, and we performed cross-validation of the DRS measurement results with the readings derived from the Corneometer and Mexameter as well as a portion of the PASI scores. Our results revealed that the erythema readings from the Mexameter were a good representation of skin oxygenated hemoglobin but not the deoxygenated hemoglobin. On the other hand, the dermatologists recruited in this study were inclined to rate higher scores on the “erythema” category as skin’s deoxygenated hemoglobin level was higher. Thus, the Mexameter derived erythema readings may not be coherent with the PASI erythema scores. Further, the Corneometer derived skin capacitance readings were well correlated to the PASI “desquamation” and “thickness” scores, while the PASI “desquamation” evaluation was a dominating factor contributing to the DRS deduced water-protein binding status. We conclude that the DRS method could be a valuable addition to existing skin capacitance/reflectance measurement systems and the PASI scoring system toward achieving a more efficient and objective clinical psoriasis vulgaris severity evaluation.

## Introduction

Skin provides several important functions, such as barrier function to preventing invasion of pathogens, thermoregulation to stabilizing body temperature, and sensation for receiving internal and external information. Once the complex physiology in skin is disturbed, the skin function would be compromised. Some skin disorders are minor, others may lead to life-threatening systemic problems. Given psoriasis vulgaris, an inflammatory disease, as an example, it is often associated with several significant comorbidities including arthritis, cardiovascular diseases, and diabetes. The diagnosis of skin disorders usually commences with clinical observations assessing skin integrity using photographic scales or non-invasive instruments. Using non-invasive instruments to evaluate skin integrity is especially valuable for the quantification of skin features that are either discernible, such as erythema, or hard to perceive, such as skin barrier function and hydration, by the naked eyes. Psoriasis vulgaris is a systemic inflammatory disease and affects more than 125 million people worldwide, and it is usually characterized by hyperkeratosis, hyperplasia of the epidermis and the dilated microvasculature in the upper dermis^1^. Dermatologists commonly employ the Psoriasis Area and Severity Index (PASI) scoring system to assess the severity of psoriasis vulgaris. The PASI scoring parameters include ratings of erythema, thickness, scaling, and area of psoriasis vulgaris, with each feature scored as 0 (null), 1 (slight), 2 (moderate) 3 (severe), or 4 (very severe). Although this rating system has been commonly used for evaluating the severity of psoriasis vulgaris, the intra- and interobserver variability of PASI assessments may exist, especially when it’s an image-based and not “live” measurements^2^.

To objectively compare the severity of psoriasis vulgaris between patients or evaluate the treatment efficacy of current therapeutic methodologies and emerging novel systemic or topical medications, researchers utilize instruments that measure transepidermal water loss and skin capacitance to investigate skin barrier function. For example, using a Tewameter, Nakajima et al. found that ceramide deficiency in the epidermis would lead to high transpeidermal water loss that would cause psoriasis vulgaris-like lesions^3^. Also, Takahashi et al. quantified skin hydration through capacitance measurements on the skin and concluded that the stratum corneum was less hydrated in psoriatic skin than in healthy skin but would revert back to normal level after treatments^4^. In addition, erythema is another dominant feature of psoriasis vulgaris. By measuring the skin reflectance at near infrared wavelength range, Li et al. quantitatively verified that the development of erythema of psoriatic lesions could be effectively reduced by applying proper topical medication^5^.

Diffuse reflectance spectroscopy (DRS) is a non-invasive optical diagnostic method that has been widely applied to investigate the physiological status of skin. In its basic configuration, a DRS system consists of a light source and light detectors to capture dermal reflectance, from which the spectroscopic features of skin are analyzed via computation models to deduce the skin properties^6–8^. Previously, we reported the use of spatially resolved DRS in quantifying microvasculature and morphology of psoriasis vulgaris^9,10^. Specifically, the increases of hemoglobin concentration in the dermis as well as the mean size of tissue scatterers induced by epidermal hyperplasia were observed in psoriasis vulgaris patients. Furthermore, we found that the compromised epidermal barrier function in psoriatic skin can be evaluated by the reflectance variation within the absorption spectrum at near-infrared wavelengths (1230–1380 nm) caused by the modification of skin bound water content.

Likewise, various commercially available systems have been applied to assist noninvasive, quantitative evaluation of the psoriasis vulgaris skin condition. Despite numerous comparisons of those systems with the PASI scoring systems, they are rarely compared on parity with DRS based systems. In this study, we measured the skin of psoriasis vulgaris patients using a Courage + Khazaka MPA 580 system and our custom-built DRS system to evaluate skin barrier function and erythema. Measurement results from these two systems were compared to each other alongside the PASI scores determined by four dermatologists for investigation of their clinical inferences and potential values in evaluating psoriasis vulgaris.

## Results and discussion

### Evaluation of skin barrier function

**Figure 1:**
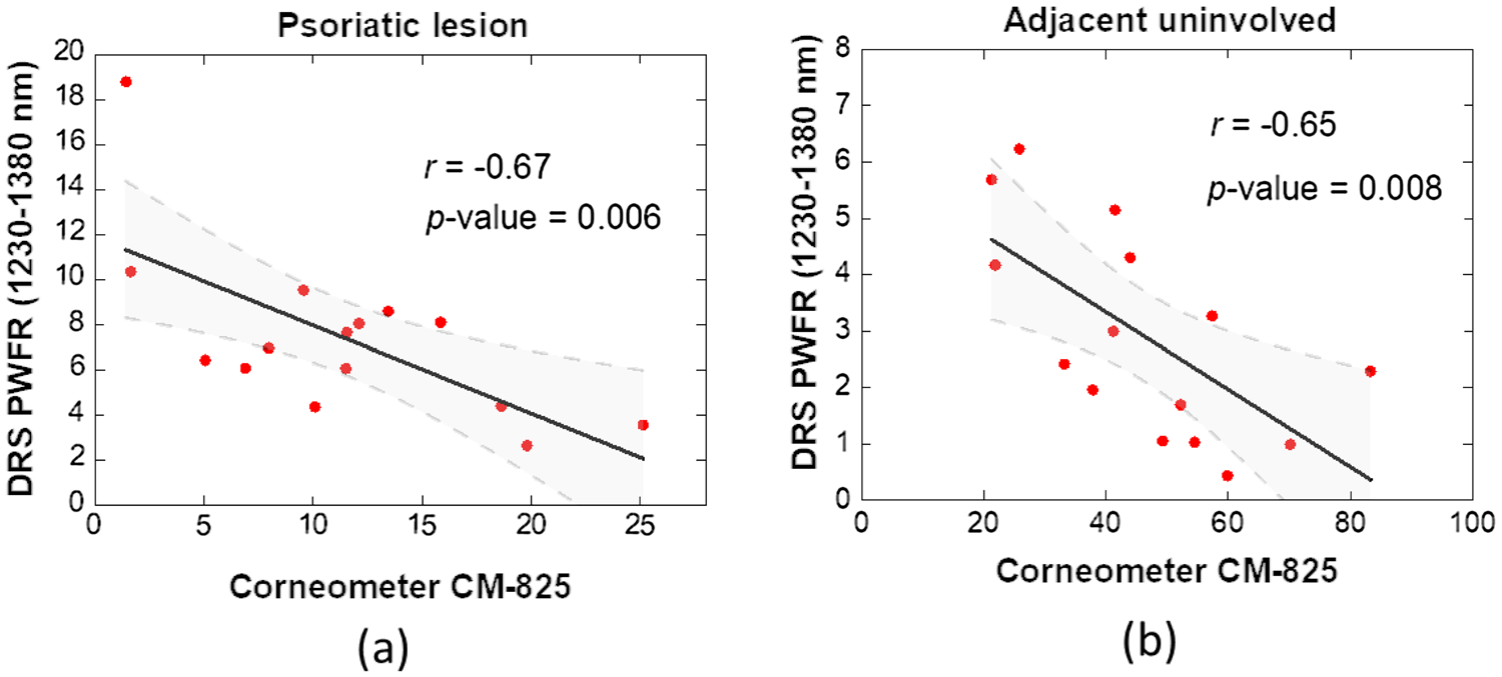
Correlation plots of the pure water fitting residuals at the first water absorption overtone obtained from the DRS system and the Corneometer hydration readings for (**a)** the psoriatic lesion sites and (**b**) their adjacent normal sites. The r values indicate the Pearson correlation coefficients. Shaded domain represents 95% confidence band. A *p*-value greater than 0.05 represents that the fitted model is not significantly different from the model *y* = constant (no correlation).

The skin barrier function of the psoriatic lesion sites and their adjacent normal sites were evaluated using our DRS system and the Corneometer CM-825, which are shown in Fig. 1a,b, respectively. For both lesion and normal sites, the PWFR values deduced from DRS were correlated decently to the values obtained with the Corneometer CM-825. Based on the working mechanism of the systems, when the water content of epidermis is low, the PWFR most likely has high values and the Corneometer settles at low values. Therefore, it can be noted in Fig. 1 that the PWFR values and the values obtained from the Corneometer have negative Pearson correlation coefficients. In addition, the Corneometer readings were in the ranges from 1.42 to 25.12 for the lesion sites and from 21.26 to 83.24 for the normal sites. The *p*-value from Student’s t-test for the Corneometer readings at the lesion and adjacent normal sites was 2.08 × 10^–6^, which conveys that the readings for the lesion site were significantly lower than those of the normal site. Takahashi et al. also utilized the Courage + Khazaka Corneometer to study the barrier function of psoriatic skin^4^. Similar to our results extracted in present study, their mean Corneometer readings for the psoriatic lesion sites and normal sites were 15.9 (ranging from 0 to 56.3) and 37.9 (ranging from 12.1 to 60.1), respectively, and the *p*-value of the two groups of readings was reported to be less than 0.01. On the other hand, the PWFR values obtained from the DRS measurements were in the ranges from 2.65 to 18.79 for the lesion site, and from 0.43 to 6.23 for the adjacent normal site. We obtained a *p*-value of 2.37 × 10^–3^ for the two data groups, verifying that the PWFR values for the lesion site were significantly higher than those for the normal site. The averages and the standard deviations of the DRS derived PWFR values and the Corneometer readings of the 15 psoriasis vulgaris patients are summarized in Table 1. Our results demonstrate the capability of the two systems in differentiating between the psoriatic lesion and the normal skin, and the readings of the two systems were properly correlated.

**Table 1:** The averages and the standard deviations of the DRS derived PWFR values and the Corneometer readings measured at the psoriatic lesion sites and their adjacent uninvolved sites of 15 psoriasis vulgaris patients.

Average/standard deviation	Corneometer® CM-825	DRS PWFR
Psoriatic lesion	11.37/6.36	7.44/3.70
Adjacent uninvolved	46.19/16.91	2.90/1.77

### Evaluation of skin erythema

**Figure 2:**
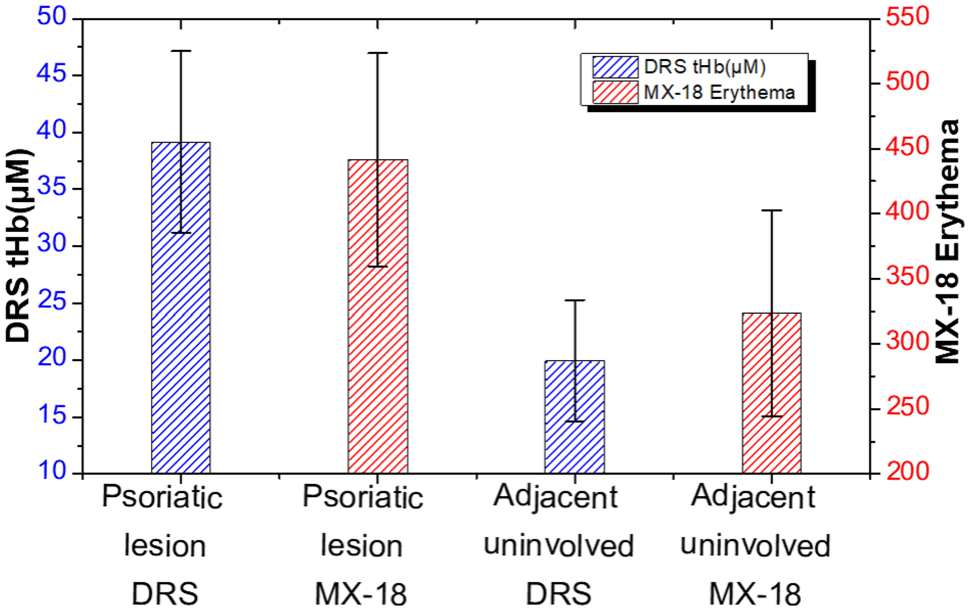
Values of total hemoglobin and erythema scores at the psoriatic lesion and uninvolved sites of 15 subjects obtained from DRS and Mexameter.

A Mexameter MX-18 and the DRS system were also used to quantify the erythema of psoriasis vulgaris skin. While the Mexameter displays the relative erythema scores of skin, the DRS system determines the average molar concentrations of oxygenated hemoglobin (O_2_Hb) and deoxygenated hemoglobin (HHb) in skin. Determination of the relative erythema scores and the total hemoglobin concentration (tHb) of psoriasis vulgaris skin of 15 subjects by the Mexameter and the DRS system, respectively, are shown in Fig. 2. It can be seen in Fig. 2 that the DRS’s tHb values at the psoriatic lesion sites were higher than those at the adjacent normal sites (*p* = 3.9 × 10^–8^). Similarly, the Mexameter-derived erythema values were higher at the lesion sites (score mean = 441.53) than at the adjacent normal sites (score mean = 323.36) (*p* = 9.4 × 10^–5^). The results of Mexameter measurement agree well with those reported by Montero-Vilchez et al., where they found the erythema scores at psoriatic lesion sites (score mean = 401.09) were statistically higher than those at normal sites (score mean = 291.12) of 38 subjects^11^. Our results shown in Fig. 2 demonstrate that Mexameter and DRS are both valid methods for distinguishing psoriasis vulgaris from the normal skin.

**Figure 3:**
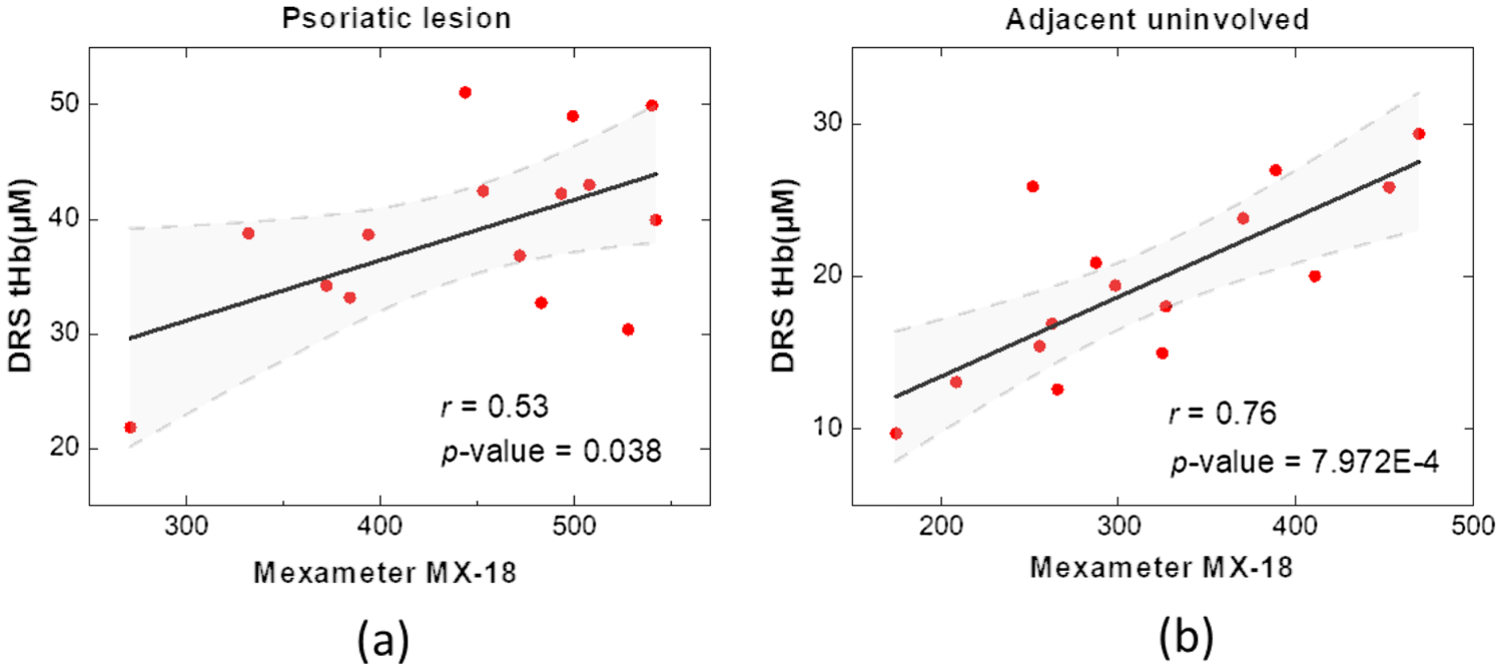
Correlation plots for total hemoglobin values and erythema scores collected from the DRS and Mexameter, respectively, at (**a**) psoriatic lesion and at (**b**) adjacent uninvolved skin. Shaded domain represents 95% confidence band. A *p*-value greater than 0.05 represents that the fitted model is not significantly different from the model *y* = constant (no correlation).

The correlation between the values of total hemoglobin deduced from our DRS system and the erythema scores obtained from the Mexameter are displayed in Fig. 3. It can be seen in Fig. 3a that measurement results of the two systems garnered at the psoriatic lesion site had a moderate correlation coefficient of 0.53, and increased to *r* = 0.76 when the measurements were performed at the adjacent healthy sites as shown in Fig. 3b.

**Figure 4:**
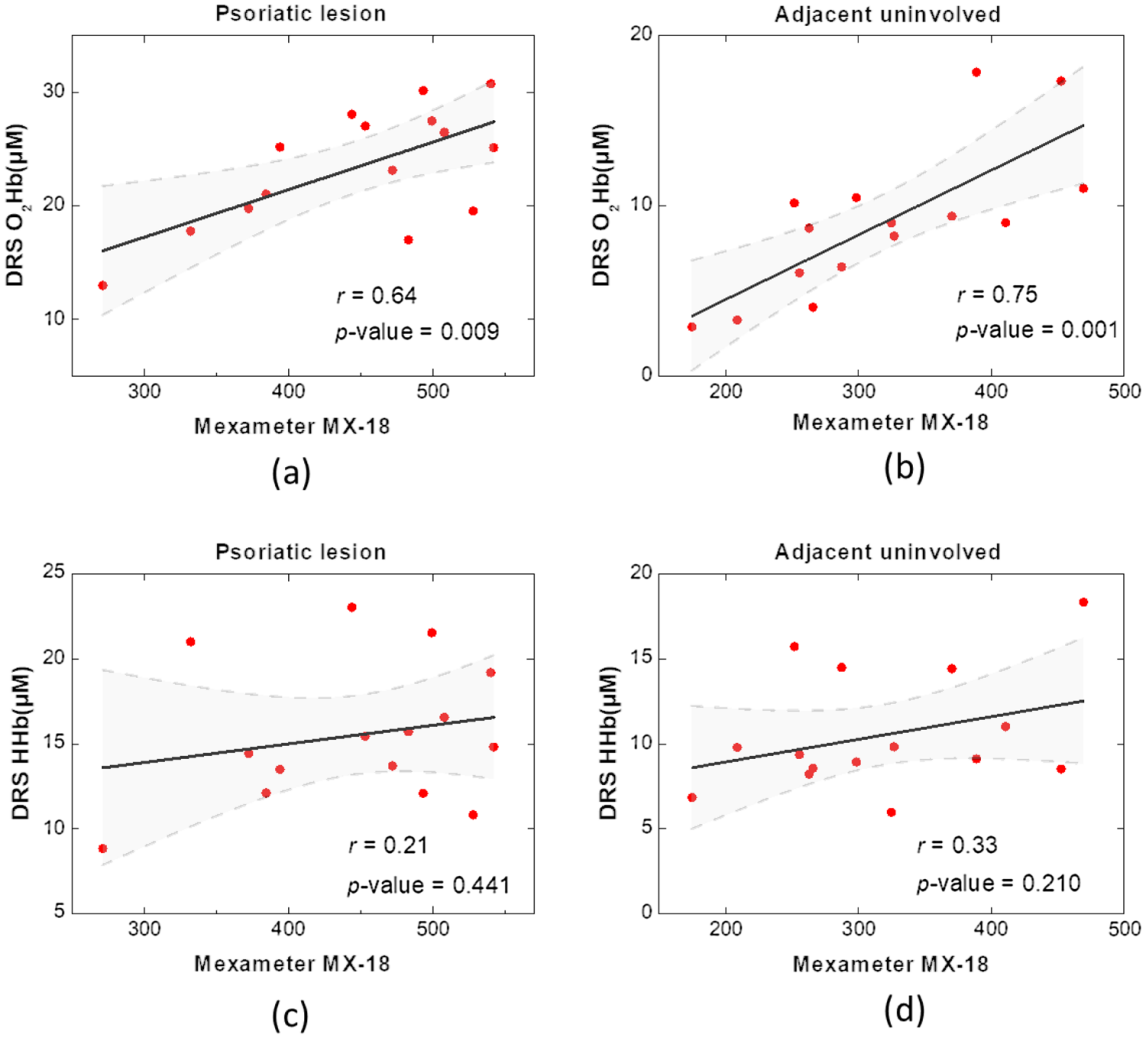
Correlation plots for the oxygenated and deoxygenated hemoglobin values and erythema scores obtained from DRS and Mexameter, respectively, at (**a**,**c**) psoriatic lesion, and at (**b**,**d**) adjacent uninvolved skin. Shaded domain represents 95% confidence band. A *p*-value greater than 0.05 represents that the fitted model is not significantly different from the model *y* = constant (no correlation).

We also conducted correlation analysis between the erythema scores collected from Mexameter MX-18 and the oxygenated/deoxygenated hemoglobin concentrations quantified from the DRS system. From Fig. 4a,b, we can observe that O_2_Hb and erythema scores have moderate correlation of *r* = 0.64 and 0.75 at the sites of the lesion and adjacent healthy skin, correspondingly. On the other hand, as shown in Fig. 4c,d, the correlation between HHb and erythema scores are weak, where the correlation coefficients were 0.21 and 0.33 at the sites of psoriatic lesion and the adjacent healthy skin, respectively. It is worth noting that, the fitted linear models (Fig. 4c,d) are not significantly different from the model *y* = constant (no correlation) (*p* = 0.441 and 0.210). Our finding suggests that while the Mexameter readings are moderately correlated to tHb values, they are more sensitive to the O_2_Hb variation than to the HHb variation. Rocha-Pereira et al. discovered that red blood cells have a limited capacity of biosynthesis and poor repair mechanisms for psoriasis vulgaris and indicated that red blood cell number could be a worsening marker of the disease^12^. On the other hand, since the proliferation of keratinocytes and the infiltration of inflammatory cells in psoriatic skin could increase oxygen consumption, hypoxia exists in the dermal capillaries and is a key factor contributing to disease progression^13–15^. Therefore, it is crucial to measure both O_2_Hb and HHb of skin for a more precise diagnosis and treatment efficacy evaluation of psoriasis vulgaris. Thus, caution should be taken when using instruments that are more sensitive only to the variation of either O_2_Hb or HHb for the diagnosis of psoriasis vulgaris and inflammation evaluation.

### Correlation between PASI scores and instruments’ numerical quantification

The lesion sites of all subjects recruited in this study were evaluated by four dermatologists using the PASI scoring system. The average scores of the three PASI severity scoring categories for all subjects are listed in Supplementary Table 1. Since the Corneometer readings and the PWFR values obtained from our DRS system are related to the local stratum corneum morphology, we performed correlation analyses between these values and the relevant PASI “thickness” and “desquamation” scores, the results of which are listed in Table 2. It can be seen in Table 2 that the Corneometer readings correlated well with the PASI “thickness” and “desquamation” scores. Using the Fisher transformation, the significance of the difference between the two correlation coefficients was *p* = 0.298 (one-tailed Student’s t-test), meaning that both skin “thickness” and “desquamation” were major factors contributing to the Corneometer readings. The induration of psoriasis vulgaris skin may be due to the edema in dermis or the abnormal keratinocyte proliferation in epidermis, and either of these two factors could lead to changes in skin capacitance. In addition, the scaling on skin can also affect the skin capacitance. Therefore, it is reasonable to interpret that the Corneometer readings had decent correlation to the PASI “thickness” and “desquamation” scores. On the other hand, the PWFR values gathered from the DRS system is an effective indicator of water-protein binding status in epidermis which directly reflects the skin barrier function^10^. Abnormal desquamation is a visible clinical manifestation of the disrupted skin barrier function. It is therefore fair to conclude about Table 2 that the PWFR was well correlated to “desquamation” scores. Interestingly, the PWFR parameter is less correlated to the “thickness” scores representing the condition of abnormal proliferation in skin. By applying Fisher transformation to the two correlation coefficients (0.48 and 0.86), the one-tailed Student’s t-test value between them was determined to be *p* = 0.029, meaning that skin “desquamation” in the PASI evaluation was the dominating factor contributing to the DRS derived PWFR values. Further studies will be carried out to investigate the applicability of simultaneous use of Corneometer readings and PWFR values for quantifying the PASI “thickness” and “desquamation” parameters independently.

**Table 2:** Pearson correlation coefficients between PASI scores (thickness and desquamation) and the values deduced from Corneometer and DRS systems of 15 psoriasis vulgaris patients.

Pearson correlation	Corneometer® CM-825	DRS PWFR
PASI thickness	− 0.68	0.48
PASI desquamation	− 0.78	0.86

The correlation coefficients between the optically derived values and the PASI “erythema” scores evaluated by four dermatologists were determined and are listed in Table 3. First, we can see that the Mexameter readings and the O_2_Hb values were moderately correlated to the PASI “erythema” scores with a same correlation coefficient of 0.51. This agrees with the data trend shown in Fig. 4 where Mexameter readings correlate better to the O_2_Hb than to HHb. Secondly, HHb values have the highest correlation coefficient to the PASI “erythema” scores among the four optical parameters listed in Table 3. This suggests that the dermatologists could be prone to give higher scores on the “erythema” category when they visually perceive the color feature of the deoxygenated hemoglobin. Nevertheless, we applied the Fisher transformation and found the one-tailed Student’s t-test value between the two correlation coefficients, 0.77 and 0.51, to be *p* = 0.131. Due to the limited sample size, we could only infer that the deoxygenated hemoglobin component of skin could be a stronger contributing factor to the PASI “erythema” scores than the oxygenated hemoglobin does. Chronic psoriasis vulgaris is very likely to introduce various comorbidities such as anemia and cardiovascular disease. It has been reported that psoriasis vulgaris triggers inflammatory response with a release of reactive oxygen species leading to enhanced red blood cell damage^16^. In addition, the level of hemoglobin oxygenation in skin was found to decrease during the progression of psoriasis vulgaris^17^. Thus, it is critical to monitor the variation of total hemoglobin in patients diagnosed with psoriasis vulgaris. On the other hand, other chronic disease with a low hemoglobin level may lead to the incidence of psoriasis vulgaris. It was shown that patients with chronic kidney disease accompanied with anemia had a significant higher incidence rate of psoriasis vulgaris than those without anemia^18^. This suggests the importance of monitoring hemoglobin level in patients of chronic disease so that proper treatment regimens could be applied to avoid psoriasis vulgaris or fend off its progression. Our finding reported here warrants the extended studies to investigate the benefit of using DRS that quantifies the total hemoglobin level for monitoring the treatment efficacy of psoriasis vulgaris.

**Table 3:** The Pearson correlation coefficients between PASI erythema scores and the values obtained from the Mexameter and DRS measurements upon 15 patients diagnosed with psoriasis vulgaris.

Pearson correlation	Mexameter® MX-18	DRS THb	DRS O_2_Hb	DRS HHb
PASI Erythema	0.51	0.74	0.51	0.77

## Conclusion

Measurement tools for skin capacitance and erythema, such as the Courage + Khazaka MPA 580 system, are commonly seen in clinical settings and are useful for quantifying the skin barrier function and erythema of psoriasis vulgaris patients. In this study, we demonstrated that by employing the wavelengths between 500 to 600 nm and between 1230 to 1380 nm, the DRS method can do the same to quantify the erythema level and the skin barrier function, and the values deduced from the DRS system and the MPA 580 system were well correlated. It was noted that the DRS elucidating the state of skin-water binding was dominantly linked to the PASI “desquamation” score, while the Corneometer readings were linked to both PASI “desquamation” and “thickness” scores. Expressly, it was found that the Mexameter was sensitive to the O_2_Hb variation but not to the HHb variation. However, the four dermatologists recruited in this study were inclined toward higher scores on the “erythema” category as skin’s deoxygenated hemoglobin level was higher. Therefore, the Mexameter readings may not be a good representation of PASI “erythema” scores. Since hypoxia and the tHb values were found to be correlated to skin inflammatory response in the progression of psoriasis vulgaris, it is thus important to monitor both O_2_Hb and HHb for a more thorough understanding of the status of skin inflammation^12,14–16^. We conclude that the DRS method can provide quantitative evaluation of skin barrier function and hemoglobin concentrations and thus could be an additional valuable tool to the current skin capacitance/erythema measurement systems and the PASI scoring system. A combinatory use of these methods could lead to a more efficient and judicious clinical evaluation of the condition of psoriasis vulgaris. We are integrating the skin capacitance measurement functionality into our DRS system and will verify the efficacy of this new system in objective assessment of the severity of psoriasis vulgaris.

## Materials and methods

### Configurations of measurement systems

#### DRS system

**Figure 5:**
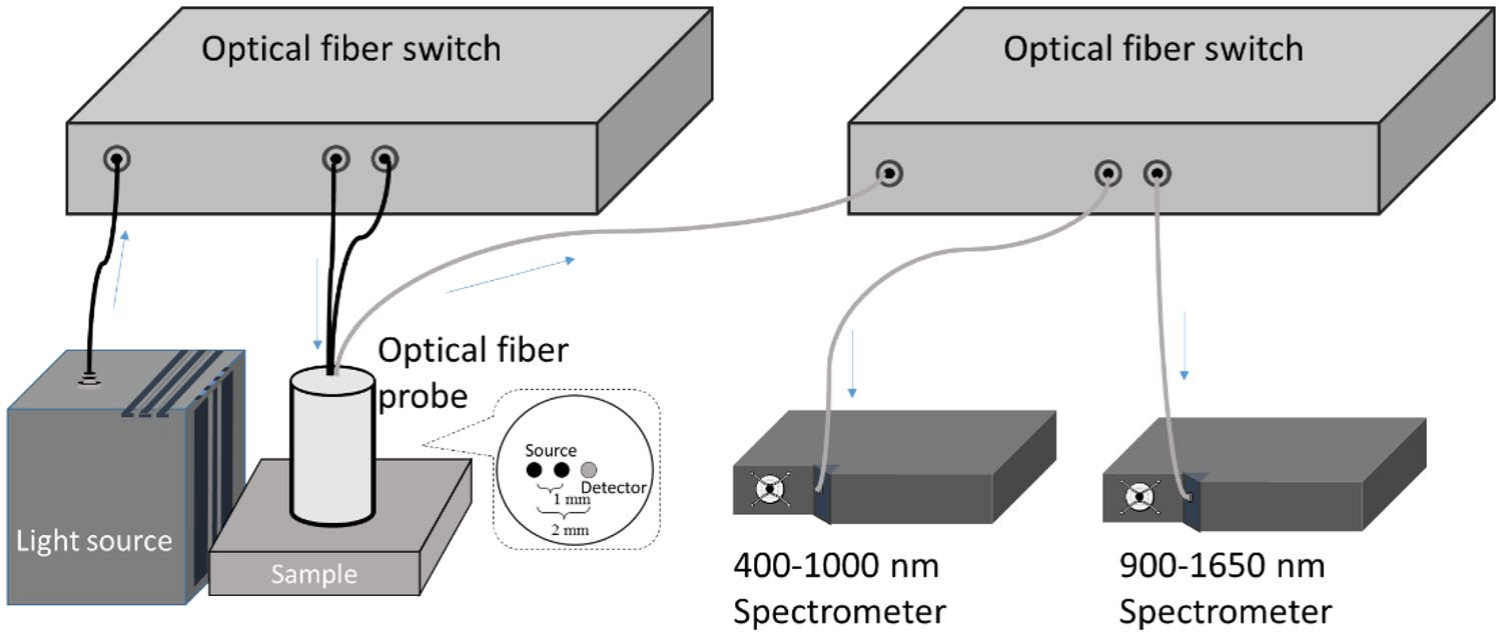
Schematic of the DRS system. Two optical switches were used in the system where one was used for switching light source to one of the two source fibers, and the other was used to switch the collected light signal to one of the two spectrometers. The fiber setup of the optical probe is shown in the inset.

In this study, we constructed a DRS system consisting of a broadband light source (EQ-99X, Energetiq, USA), two spectrometers (QE65000 and NIR QUEST, Ocean Optics, USA), two 1 × 2 optical fiber switches (Piezosystem Jena, Germany), and a custom optical fiber probe as shown in Fig. 5. The fiber probe is harnessed with three aligned fibers of diameter and numerical aperture of 400 μm and 0.22, respectively, for light delivery and collection. A schematic of the fiber arrangement is shown in the inset of Fig. 5. A custom routine was developed to operate the system and record skin diffuse reflectance at the two source-to-detector separations (SDSs) within the wavelength range from 500 to 1600 nm for the determination of absorption and reduced scattering coefficients of skin.

#### Courage + Khazaka MPA 580 system

The reference commercial system employed in this study was a Multi Probe Adapter system from Courage + Khazaka (model number MPA 580, Courage + Khazaka electronic GmbH, Germany). We attached a Mexameter MX-18 probe and a Corneometer CM-825 probe to the main system for measuring skin erythema and hydration, respectively. The Corneometer CM-825 probe measures the capacitance of skin and reckon high capacitance reading when skin is well hydrated, due to the fact that water has a higher dielectric constant than most other substances in skin. Corneometers have been used as a noninvasive method to quantify the skin barrier function. The Mexameter MX-18 probe measures the skin reflection at the wavelengths of 568 nm and 660 nm which correspond to the absorption peaks of hemoglobin. As suggested in the user manual, the measurement values of the Mexameter MX-18 are relevant to the skin absorption and are positively correlated to skin erythema.

### Theoretical model

#### Skin absorption and reduced scattering coefficients

The skin reflectance collected at SDS of 1 and 2 mm by the DRS system were converted to absorption and reduced scattering coefficients using an artificial neural network (ANN) model. The construction and validation of this model were described in detail elsewhere^19^. In short, an ANN model was trained with diffuse reflectance computed from Monte Carlo simulations to determine the spectral information of tissue absorption and reduced scattering from the measured diffuse reflectance spectra. Specifically, the model was used to recover the skin absorption and reduced scattering spectra in the wavelength range from 500 to 1380 nm, from which we used a wavelength span of 500 nm to 600 nm to quantify the hemoglobin level of skin and another wavelength range from 1230 to 1380 nm to investigate the state of dermal water-protein binding state^7,10^.

#### Level of skin hemoglobin and pure water-fitting residual

The derived skin absorption spectra in the wavelength range of 500–600 nm were fit linearly to the absorption spectra of oxygenated hemoglobin and deoxygenated hemoglobin for extraction of the skin hemoglobin concentrations, including O_2_Hb, HHb, and their sum tHb, based on Beer’s law^20–22^. Previously, we reported the use of this method to quantify the hemoglobin concentrations of normal, keloidal, as well as psoriatic skin^7,9^. Additionally, we verified that the subtle disparity between the spectral contents of skin absorption and pure water within the wavelength span of 1230 to 1380 nm, at the first water absorption overtone, could be used to investigate the affinity status between the protein and water that linked to the skin barrier function. Specifically, we used a curve fitting method of the “lsqcurvefit” function in MATLAB to determine the best fit of the absorption spectrum of pure water to the skin absorption spectra. The fitting residual returned from the function was divided by the number of spectral data points, and these values were then multiplied by 100 and defined as the “pure water fitting residual” (PWFR), which are displayed in Fig. 1.

### Protocol of clinical study

In the present study, we recruited 15 subjects who had been diagnosed with psoriasis vulgaris by dermatologists at National Cheng-Kung University Hospital, Taiwan. The protocol was approved by the Institutional Review Board of National Cheng Kung University Hospital, Taiwan (No. ER-100-332). Written informed consents were obtained from all the subjects in the study. The study was conducted in accordance with relevant guidelines/regulations as well as the latest revision of the Declaration of Helsinki. For each subject with psoriasis vulgaris, a psoriatic lesion site selected by dermatologists was measured, in addition to an adjacent normal site at least 2 to 3 cm apart from the edge of lesion. Three parameters of PASI scoring system, namely, erythema, thickness, and desquamation, were rated based on the photos of lesions and the average of the scores from four dermatologists are listed in Supplementary Table S1.

### Statistical analysis

OriginPro2017 software (OriginLab, USA) was employed to calculate the Pearson’s correlation coefficient (*r*) of two data series. In addition, the software also provides the *p*-value to determine whether the fitted model is significantly different from the model *y* = constant (no correlation). The statistical significance was defined at *p* < 0.05. To determine whether two correlation coefficients were significantly different, the Fisher transformation was used to transform the sampling distribution of Pearson’s correlation coefficients to normal distribution. The transformed correlation coefficients were used to compute the “z” value and *p*-value, and the statistical significance was defined at *p* < 0.05.

## Supplementary Information

Supplementary Information.

## Data Availability

The datasets used and/or analysed during the current study are available from the corresponding author on reasonable request.
