# Supplementary material for: Investigating the clinical implication of corneometer and mexameter readings towards objective, efficient evaluation of psoriasis vulgaris severity
Source: Sci Rep. 2022 May 6;12:7469. doi: 10.1038/s41598-022-11573-2 (PMC9076632; doi:10.1038/s41598-022-11573-2)
Supplement: Supplementary file 1 — Supplementary Information. [file 41598_2022_11573_MOESM1_ESM.docx]

# Supplementary Information

# Table S1. The average of scores rated by four dermatologists of the erythema, thickness, and desquamation of the psoriatic lesion sites of 15 patients.

| Subject | Score | | | Subject | Score | | |
| --- | --- | --- | --- | --- | --- | --- | --- |
|  | Erythema | Thickness | Desquamation |  | Erythema | Thickness | Desquamation |
| P01 | 1.75 | 2.50 | 1.25 | P09 | 2.00 | 0.75 | 1.00 |
| P02 | 1.25 | 2.00 | 1.75 | P10 | 1.75 | 0.75 | 1.25 |
| P03 | 1.75 | 2.50 | 2.75 | P11 | 2.50 | 1.25 | 1.75 |
| P04 | 0.50 | 0.50 | 0.75 | P12 | 3.25 | 2.00 | 3.00 |
| P05 | 2.75 | 2.50 | 1.00 | P13 | 0.75 | 2.25 | 2.50 |
| P06 | 2.50 | 1.75 | 1.50 | P14 | 2.25 | 1.00 | 1.50 |
| P07 | 1.50 | 1.25 | 0.75 | P15 | 1.25 | 0 | 0.25 |
| P08 | 2.25 | 0.75 | 1.00 |  |  |  |  |
